# Complex Involvement of Interleukin-26 in Bacterial Lung Infection

**DOI:** 10.3389/fimmu.2021.761317

**Published:** 2021-10-28

**Authors:** Karlhans F. Che, Magnus Paulsson, Krzysztof Piersiala, Jakob Sax, Ibrahim Mboob, Mizanur Rahman, Rokeya S. Rekha, Jesper Säfholm, Mikael Adner, Peter Bergman, Lars-Olaf Cardell, Kristian Riesbeck, Anders Lindén

**Affiliations:** ^1^ Unit for Lung and Airway Research, Institute of Environmental Medicine, Karolinska Institutet, Stockholm, Sweden; ^2^ Karolinska Severe Chronic Obstructive Pulmonary Disease (COPD) Center, Department of Respiratory Medicine and Allergy, Karolinska University Hospital Solna, Stockholm, Sweden; ^3^ Clinical Microbiology, Department of Translational Medicine, Faculty of Medicine, Lund University, Malmö, Sweden; ^4^ Division of Infection Medicine, Department of Clinical Sciences, Faculty of Medicine, Lund University, Lund, Sweden; ^5^ Department of Infectious Diseases, Skåne University Hospital, Lund, Sweden; ^6^ Division of Ear Nose and Throat (ENT) Diseases, Department of Clinical Sciences, Intervention and Technology, Karolinska Institutet, Stockholm, Sweden; ^7^ Department of Ear Nose and Throat (ENT) Diseases, Karolinska University Hospital, Stockholm, Sweden; ^8^ Unit of Integrative Toxicology, Institute of Environmental Medicine (IMM), Karolinska Institutet, Stockholm, Sweden; ^9^ Division of Clinical Microbiology, Department of Laboratory Medicine, Karolinska Institutet, Stockholm, Sweden; ^10^ Institute of Environmental Medicine, Karolinska Institutet, Stockholm, Sweden; ^11^ Centre for Allergy Research, Karolinska Institutet, Stockholm, Sweden; ^12^ Immunodeficiency Unit, Department of Infectious Disease, Karolinska University Hospital, Stockholm, Sweden

**Keywords:** IL-26, IL-10 and Th17 cytokines, bacteria pneumonia, bacterial killing, macrophages, neutrophils, lung tissue analysis and transcription, myeloperoxidase and elastase

## Abstract

Pneumonia is a global cause of mortality, and this provides a strong incentive to improve the mechanistic understanding of innate immune responses in the lungs. Here, we characterized the involvement of the cytokine interleukin (IL)-26 in bacterial lung infection. We observed markedly increased concentrations of IL-26 in lower airway samples from patients with bacterial pneumonia and these correlated with blood neutrophil concentrations. Moreover, pathogen-associated molecular patterns (PAMPs) from both Gram-negative and -positive bacteria increased extracellular IL-26 concentrations in conditioned media from human models of alveolar epithelial cells, macrophages, and neutrophils *in vitro*. Stimulation with IL-26 inhibited the inherent release of neutrophil elastase and myeloperoxidase in unexposed neutrophils. This stimulation also inhibited the expression of activity makers in neutrophils exposed to *Klebsiella pneumoniae.* In addition, priming of human lung tissue *ex vivo* with exogenous IL-26 potentiated the endotoxin-induced increase in mRNA for other cytokines involved in the innate immune response, including the master Th17-regulator IL-23 and the archetype inhibitory cytokine IL-10. Finally, neutralization of endogenous IL-26 clearly increased the growth of *Klebsiella pneumoniae* in the macrophage culture. These findings suggest that IL-26 is involved in bacterial lung infection in a complex manner, by modulating critical aspects of innate immune responses locally and systemically in a seemingly purposeful manner and by contributing to the killing of bacteria in a way that resembles an antimicrobial peptide. Thus, IL-26 displays both diagnostic and therapeutic potential in pneumonia and deserves to be further evaluated in these respects.

## Introduction

Bacterial lung infection affects both children and adults worldwide (1–5). Although prevention has improved due to improved health care, antibiotics and vaccinations, pneumonia remains a major cause of premature death in many parts of the world (1–5). To facilitate the development of more effective therapies, we need to improve our understanding of the innate immune response during bacterial lung infection.

During the last decade, the neutrophil-mobilizing cytokine interleukin (IL)-26 has emerged as an important player in the innate immune response of humans (6, 7). Briefly, this cytokine is abundant in the airways of healthy humans, and its concentrations are further enhanced after intrabronchial exposure to endotoxins from Gram-negative bacteria (6). In addition, these concentrations of IL-26 are substantially enhanced in long-term smokers who are colonized by bacterial pathogens (8). Moreover, there is a positive correlation between IL-26 and leukocyte and neutrophil cell concentrations in the airways of healthy subjects, with or without prior endotoxin exposure, as well as in patients with chronic obstructive pulmonary disease (COPD) (8, 9). However, the corresponding associations of local IL-26 and systemic leukocyte concentrations have not yet been characterized.

With reference to cellular sources, it is known that IL-26 is produced by myeloid and mesenchymal cells that reside in the airways, including T lymphocytes (CD4^+^, CD8^+^ plus Th17 cells) (9), alveolar macrophages (8, 9), bronchial epithelial cells (10), and lung fibroblasts (9, 11). However, there is no information available on the production of IL-26 in alveolar epithelial cells or neutrophils, cells that are strategically located during bacterial lung infection, in the peripheral airways and lung parenchyma, respectively.

There is evidence that IL-26 promotes the innate immune respone to endotoxin in the lungs of mice (12), and that IL-26 triggers the innate immune response against pathogen-associated molecular patterns (PAMPS) of Gram-negative bacteria both *in vitro* and *in vivo* (8, 9, 12–15). Notably, several experimental findings indicate that IL-26 *per se* inhibits the growth of the Gram-negative species *Klebsiella pneumoniae*, *Pseudomonas aeruginosa*, and *Escherichia coli*; as well as of the Gram-positive species *Staphylococcus aureus* (13, 16). The existing data shows that recombinant human (rh) IL-26 kills *P. aeruginosa* through disruption of the bacterial cell membrane by pore formation leading to ion leakage (13). However, despite the evidence that IL-26 may be involved in the innate immune response in the lungs, its involvement in human patients with bacterial lung infection has not yet been characterized. Here, we hypothesized that IL-26 is critically involved in bacterial lung infection, that local cells being present in this condition, such as alveolar epithelial cells, neutrophils, and macrophages, produce local IL-26 and that this IL-26 modulates the innate immune response and kills bacteria as well.

To evaluate our hypothesis, we investigated the involvement of IL-26 in bacterial pneumonia in human subjects and the immunological mechanisms behind this involvement. We did this by quantifying protein concentrations of IL-26 in lower airway samples from patients with bacterial pneumonia and characterized their relationship with leukocyte and neutrophil concentrations in peripheral blood. We also examined the production of IL-26 production in human models of alveolar type II epithelial cells, neutrophils, and macrophages in response to bacterial stimuli. Furthermore, we demonstrated the effects of exogenous IL-26 on functional attributes of neutrophils and macrophages, as well as on innate immune signaling in human lung tissue. Finally, we assessed the effect on bacterial killing by neutralizing endogenous IL-26 in a macrophage model *in vitro*. Considering the results altogether, we obtained evidence that IL-26 is involved in bacterial lung infection in a complex manner. In addition to what is previously known about cellular sources, we show that IL-26 released by alveolar epithelial cells, neutrophils and macrophages, that it modulates innate immune responses in neutrophils, and macrophages; that it potentiates the transcription of key pro- and anti-inflammatory cytokines in human lung tissue during bacterial exposure; and that it directly contributes to the killing of bacteria.

## Materials and Methods

### Human Study Materials

For both pneumonia materials addressed below, we recruited subjects with clinical signs (≥ 2) of pneumonia including: altered body temperature (> 38.5°C or < 36.5°C), leukocyte concentrations (> 12,000 cells or < 4,000 cells per μL), purulent endotracheal aspirate or sputa, and radiographic pulmonary infiltrate.

*The Pneumonia 2016 material.* The subjects were recruited and sampled from 2015 to 2016. Here, lower airway samples were harvested as bronchoalveolar lavage samples (BAL: 3 x 50 mL phosphate buffered saline (PBS), from 14 pneumonia patients during bronchoscopy. Because of limited access, we accepted BAL samples from both ventilated (*n*=7) and non-ventilated *(n* =7) patients with pneumonia. For these pneumonia samples, we established control samples using BAL samples from healthy volunteers who were not ventilated, referred to as control subjects. The bronchoscopy was performed by a specialist in intensive care (Flexible bronchoscopy; Olympus Optical Co) according to local clinical practice (17). Briefly, during BAL, the PBS was instilled in the lung segments showing the most severe signs of disease, based on visual assessment. The BAL samples were then harvested, filtered, and centrifuged (300 x g followed by 900 x g). The established cell free-free BAL fluid samples were then stored at -80°C and utilized later for cytokine analyses. The principal clinical and laboratory characteristics (demographics) of this material are presented in **Table 1**.

**Table 1:** Pneumonia 2016 material.

Subject characteristics	Control subjects	Pneumonia patients
Subjects (*n*)	7	14
Sex (female/male)	2/5	6/8
Bacteria (Yes/No)	0/7	12/2
Fungal growth (Yes/No)	0/7	3/11
Age (Years)	49 (41–74)	74 (39–83)
Mechanical ventilator (Yes/No)	0/7	7/7
Nosocomial pneumonia (Yes/No)	0/7	11/3
Community acquired pneumonia (Yes/No)	0/7	3/11
Current smokers	0	0
Former smokers	0	3
Nonsmokers	7	9
C-reactive protein	0.64 (0.44 – 1.6)	69.5 (8.6–194)
Leukocytes (x10^9^/L)	5.2 (4 – 7)	9.85 (1.1–17.5)
Neutrophils (x10^9^/L)	3 (2.3 – 3.6)	9.1 (3.4–15.6)
Antibiotics (Yes/No)	0/7	14/0
Systemic steroids (Yes/No)	NA	0/14
Inhaled steroids (Yes/No)	NA	2/12
Fi02 (L/min) %	NA	NA
COPD (Yes/No)	0/7	1/13
Other pulmonary diseases (Yes/No)	0/7	2/12
Malignant disease (Yes/No)	0/7	3/11
Sepsis (Yes/No)	0/7	10/4
Radiographic infiltrates (Yes/No)	0/7	11/03
Duration of symptoms	–	13.5 (1–34)
Purulent secretion (Yes/No)	0	8/4

Data for study subjects donating bronchoalveolar lavage (BAL) samples. Notably, in the control subjects, data on systemic or inhaled steroids was not available (NA). Moreover, data on the fraction of inspired oxygen (Fi02) in the control and patient group was NA.

*The Pneumonia 2017 material.* The subjects were recruited and sampled during 2017. Here, lower airway samples were harvested as bronchial wash samples (BW: 2 x 10 mL PBS) in 8 pneumonia patients during bronchoscopy. For this material, we harvested all the BW samples from pneumonia patients who were being ventilated (except for 1 patient). For these pneumonia samples, we established control samples using BW samples from patients who were ventilated for reasons other than pneumonia or other lung diseases (*n*=12), referred to as control patients. The bronchoscopy was performed by a specialist in intensive care (Flexible bronchoscopy; Olympus Optical Co.) according to local clinical practice (17). Briefly, the PBS was instilled in the lung segments showing the most severe signs of disease. The BW samples were handled as described in the BAL samples and the cell-free BW fluid was stored at -80°C for later cytokine analyses. The principal clinical and laboratory characteristics (demographics) of this material are presented in **Table 2**.

**Table 2:** Pneumonia 2017 material.

Subject characteristics	Control patients	Pneumonia patients
Subjects (*n*)	12	8
Sex (female/male)	9/3	1/7
Bacterial growth (Yes/No)	12/0	7/1
Fungal growth (Yes/No)	0/12	0/8
Age (Years)	52 (21–82)	66 (27–75)
Mechanical ventilator (Yes/No)	12/0	7/1
Nosocomial pneumonia (Yes/No)	0/12	4/4
Community acquired pneumonia (Yes/No)	0/12	4/4
Current smokers	1	2
Former smokers	4	2
Nonsmokers	6	3
C-reactive protein (mg/L)	2.75 (0–66)	89.5 (29–448)
Leukocytes (x10^9^/L)	6.6 (5.2–11.1)	10.25 (4.6–19.3)
Neutrophils (x10^9^/L)	3.9 (2.4–6.4)	7.9 (2.7–19)
Antibiotics (Yes/No)	7/5	8/0
Systemic steroids (Yes/No)	0/12	2/6
Inhaled steroids (Yes/No)	0/12	5/3
Other immunosuppressants	0/12	0/8
Arteria oxygen Sat (%)	96.5 (95 – 100)	96 (87–99)
Fi02 (L/min) %	NA	30 (25–50)
COPD (Yes/No)	0/12	2/6
Other pulmonary diseases (Yes/No)	0/12	0/8
Diabetes Mellitus (Yes/No)	0/12	1/7
Malignant disease (Yes/No)	0/12	1/7
Sepsis (Yes/No)	0/12	7/1
Radiographic infiltrates (Yes/No)	0/12	6/3
Duration of symptoms	–	4 (2–8)
Purulent secretion (Yes/No)	0/12	3/5

Data for study subjects donating bronchial wash (BW) samples. Smoking status was not known for 1 subject in the group of control patients and for 1 subject in the group of pneumonia patients. Moreover, in the control group, data on the fraction of inhaled oxygen (Fi02) was not available (NA).

Some data from the Pneumonia 2016 and 2017 materials have been used in another publication with a scientific focus that was different from the current one (17). However, the data on IL-26 has not been utilized before.

*The Human Lung Tissue 2016 material.* Lung tissue samples were obtained from patients undergoing lobectomy due to cancer, at the Department of Thoracic Surgery (Karolinska University Hospital) from 2013 to 2015 The demographics of the donor patients are summarized in **Table 3**. During the preparation of these tissue samples, care was taken to select morphologically normal sections of the lobar lung tissue.

**Table 3:** The lung tissue material (2015).

Subject characteristics	Tissue donors
Subjects (*n*)	8
Sex (female/male)	4/4
Current smokers/Nonsmokers	2/6
Former smokers	3/5
Never smokers	3/8
C-reactive protein (mg/L)	2 (1–8)
Blood leukocytes (x10^9^/L)	6.15 (5.2–8.2)
COPD (Yes/No)	0/8
Other pulmonary diseases (Yes/No)	1/7

Data for study subjects donating lung tissue samples.

An average of 30 mg of lung tissue from each subject was cultured overnight in Roswell Park Memorial Institute (RPMI) 1640 medium in the presence of rhIL-26 (100 ng/mL), with or without the exposure to endotoxin (100 ng/mL) in 6-well plates. The tissue pieces where then stored in RLT buffer (Qiagen) at -80°C for Next Generation RNA Sequencing (NGRS).

### Microbiology

Protected brush specimens were collected in parallel with the BW or BAL samples during bronchoscopy. These samples were then analyzed according to standard procedures at the Clinical Microbiology laboratory (Laboratory Medicine Skåne, Lund, Sweden) using microbiology protocols according to local standards. Briefly, these analyses included aerobic cultures, MALDI-TOF, and identification of bacterial DNA. The different bacterial species isolated in the BAL and BW of the patients and their frequencies are presented in **Supplementary Table S1**. Of note, purulent secretion was determined visually. Sepsis was determined and classified according to the Sepsis 2 definition (18).

### Cells and Reagents

#### Alveolar Epithelial Cell Line and Human Primary Cells

We purchased A549 cells, a human model of Type II alveolar epithelial cells, from the European Collection of Authenticated Cell Cultures (ECACC) and cultured these in fetal bovine serum (FBS 10%) in Ham’s F-12K (Kaighn’s) medium supplemented with penicillin-streptomycin (100 µg/mL) (ThermoFisher Scientific). Prior to stimulation, cells were subcultured (seeded) in 24-well plates (Sigma Aldrich) until cells attained semi-confluency (80%). The A549 epithelial cells were then starved in FBS (1%) medium overnight, and subsequently stimulated with the TLR-1/2 ligand Pam3CSK4, the TLR4 ligand lipopolysaccharide (LPS; endotoxin from *E coli*), the TLR5 ligand flagellin and the TLR2/6 ligand Pam2CGDPKHPKSF, respectively (0.1 µg/ml or 1 µg/ml for all PAMPs). Stimulations (3, 6, and 24 h) took place in fresh FBS (1%) medium. The conditioned media were harvested, centrifuged (1,500 rpm for 10 min) and the cell-free conditioned media stored at -80°C prior to cytokine measurements. The adherent cells were used for assessment of mRNA.

Human blood neutrophils were isolated from whole blood of healthy donors that was purchased from the Blood Central (Karolinska University Hospital, Sweden). These neutrophils were isolated using the human MACSxpress Whole Blood Neutrophil Isolation Kit according to the manufacturer’s instructions (**Miltenyi Biotec**). The neutrophils were stimulated (3, 6 and 18 h) with endotoxin (100 ng/ml), in a medium of each blood donor´s own plasma (10%) diluted in RPMI 1640 (ThermoFisher Scientific). Moreover, the neutrophils were also stimulated (3 h) with *K. pneumoniae* at different multiplicity of infections (MOI: 0.001, 0.01, 0.1, 1, 10 and vehicle). Cell-free conditioned media were then harvested for cytokine measurements. In separate experiments, neutrophils were stimulated with rhIL-26 protein (10 and 50 ng/mL) and exposed to endotoxin (100 ng/mL), or *K. pneumoniae* (MOI; 0.001 or 0.01) simultaneously in culture (3, 6 and 18 h, at 37°C). The cell-free conditioned media were harvested and frozen (-80°C) for cytokine measurements and cells were subsequently stained for flow cytometry analyses.

Peripheral blood mononuclear cells (PBMC) were isolated from buffy coats originating from healthy donors purchased from the Blood Central (Karolinska University Hospital) and processed through density gradient centrifugation in Ficoll-Paque^™^ (GE Healthcare Biosciences). PBMCs (10 x 10^6^ in total) were plated in 6-well plates and incubated at 37°C in 5% CO_2_ in serum-free RPMI 1640 for 2 h. The nonadherent cells were washed off and the adherent cells (~10^6^ monocytes/well) were incubated in culture medium (10% FBS), supplemented with rh granulocyte macrophage colony stimulating factor (rhGM-CSF; 5 ng/mL; R&D Systems Europe) in the absence of antibiotics. Cells were differentiated for 6-7 days during which half of the culture medium (1 mL) was being replaced every 2^nd^ day with fresh culture media (1 mL) containing twice the concentration of the rhGM-CSF. To prepare for cell exposure to live *K. pneumoniae* (American Type Culture Collection (ATCC) strain 25955), frozen aliquots of bacteria were thawed, streaked on blood agar plates, and incubated overnight at 37°C. A colony of the activated *K. pneumoniae* was cultivated (37°C on a shaker [200 rpm]) in liquid broth to attain a logarithmic phase of growth at 1.5-2 h. The bacteria were washed (3 x) with PBS (1500 x g, for 5 min) and resuspended in PBS. The concentration (i.e., turbidity/optical density) was thereafter measured (600 nm wavelength) using the GENESYS Spectrophotometer (ThermoFisher Scientific). Macrophages were thereafter exposed (37°C in 5% CO_2_ in 10% FBS) to *K. pneumoniae* at different MOI (0.001, 0.01, 0.1, 1, 10 and vehicle) for 3 h. The cell-free conditioned media were harvested and stored (-80°C) for cytokine measurements and the adherent cells were used for mRNA analyses. In separate experiments, the MDMs were treated (37°C in 5% CO_2_ in 10% FBS) with different concentrations of rhIL-26 (5, 10 or 50 ng/m: R&D Systems Europe) or vehicle. The cell-free conditioned media were then harvested for cytokine measurements. The cells that were primed with rhIL-26 were thereafter washed (3x, in PBS) and exposed to *K. pneumoniae* at MOI 5 for 30 min. Cell-bound bacteria were killed using gentamicin (25 µg/mL, 30 min at 37°C) and removed by washing 3 times. The cells were then cultured for 4 h in fresh culture medium (10% FBS in RPMI). Cell-free conditioned media were harvested for cytokine measurements, whereas the adherent cells were lysed (1% Triton ™ X-100 (Sigma-Aldrich). The lysates were serially diluted and plated on blood agar overnight. Colony forming units (CFU) were counted manually and the data presented as percentage of growth of bacteria in relation to uptake. We then performed another series of experiments, in which we simultaneously treated cells with a neutralizing anti-IL-26 monoclonal IgG1 antibody (1 and 10 μg/mL) or the corresponding IgG1 isotype control (1 and 10 µg/mL) (both from R&D Systems Europe) and *K. pneumoniae* (MOI 0.01) in culture (3 and 6 h, at 37°C). The cell-free conditioned media were thereafter harvested for cytokine measurements. The cells were treated with gentamicin (25 μg/mL for 30 min at 37°C) and washed (3 x) with PBS followed by lysis in 1% Triton. The lysates were diluted in a serial manner and plated on blood agar overnight. Bacterial CFUs were counted manually. Similar parallel experiments were performed and used for the assessment of mRNA.

### Quantification of Extracellular Protein Concentrations

#### IL-26

An IL-26 ELISA (Cusabio, Nordic Biosite) was performed in accordance with the manufacturer’s instructions. Briefly, diluted samples and standards were added in duplicates to plates and incubated for 2 h at 37°C in 5% CO_2._ The biotin-conjugated detection antibody was added for 1 h followed by the avidin-conjugated Horseradish-Peroxidase (1 h) and developed with tetramethylbenzidine (TMB). The optical density was measured (450nm) using a microplate reader (Model Spectra Max 250, Molecular Devices). Notable, the detection rage of the human IL-26 was 62.5 pg/ml-4000 pg/ml. However, the minimum detectable dose is typically less than 15.6 pg/ml as stated by the manufacturer.

#### IL-6, IL-8, IL-10, Neutrophil Elastase, and Myeloperoxidase

The assays were performed according to the manufacturer’s protocol (DuoSet kits, R&D Systems, Europe). Ninety-six well plates (Nunc, Sigma-Aldrich) were coated overnight at room temperature (RT) with capture antibodies for IL-6 (2 µg/mL), IL-8 (4 µg/mL), IL-10 (2 µg/mL), NE (4 µg/mL) and MPO (4 µg/mL). The plates were then washed (3x), and the unspecific binding sites blocked with 1% BSA in PBS for app. 1 h. Plates were washed and incubated with the diluted samples and standards for 2 h at RT. Plates were again washed and incubated with the respective detection antibodies for IL-6 (50 ng/mL), IL-8 (20 ng/mL), IL-10 (75 ng/mL), NE (250 ng/mL) and, MPO (50 ng/mL). Plates were washed and incubated with TMB substrate solution and stopped with H_2_S0_4_. The optical density was measured (450nm) using a microplate reader (Multiskan Ex, microplate reader, ThermoFisher Scientific).

### Analysis of Different mRNAs

#### Isolation of Total RNA

Total RNA was isolated from the cell lysate using RNeasy plus Mini kit or the RNeasy mini kit, Hilden (Qiagen) according to the manufacturer’s protocol. The quality and the concentrations were determined using a spectrophotometer (Nanodrop, Bergman Labora)

#### cDNA Synthesis

The synthesis of cDNA was performed using a High-capacity RNA-to-cDNA kit (ThermoFisher Scientific) or the iScript™ Reverse Transcription super mix (Bio-Rad) according to the manufacturer’s protocol. A maximum of 1 µg of RNA was used for the reverse transcription process in the ThermoFisher Scientific thermal cycler or the Bio-Rad MyCycler. We utilized Fast SYBR green master mix (ThermoFisher Scientific) and the amplification measured using the 7500 Fast Real-Time PCR system (ThermoFisher Scientific). We also utilized the BIO-RAD iTaq Universal SYBR^®^ Green Supermix Protocol and the amplifications measured using the CFX96 Touch Real-time PCR Detection system (BIO-RAD). The primers were designed using the NCBI Primer-BLAST online software (https://www.ncbi.nlm.nih.gov/tools/primer-blast/) and synthesized by Cybergene or Eurofins Genomics. The reaction mix contained 4ng cDNA, 5 pmol each of forward and reverse primers, RNase-free water and SYBR green mix. Data were normalized with reference to the house keeping gene β-actin, and the relative quantification (fold-change) was obtained using the cycle threshold (Ct) according to the 2**^-ΔΔCT^**. Data was then presented as mRNA levels.

#### Next Generation RNA Sequencing

This analysis was performed at the Bioinformatics and Expression Analyses (BEA) core facility at Karolinska Institute. RNA was extracted according to RNeasy plus mini protocol according to the manufacturer’s protocol (Qiagen). The quantity and quality of the RNA was determined using an Agilent Bioanalyzer (Agilent Technologies) before sequencing. The RNA was labelled, hybridized, washed, and scanned, according to the manufacturer’s instructions (Illumina technology). The data was then analyzed and filtered such that only the genes that displayed 2-log-fold change in transcription levels were presented.

### Flow Cytometry Analyses

After stimulation, human neutrophils were washed and stained with fluorochrome-conjugated mouse anti-human Abs (BD Bioscience) targeting surface antigens including CD11b -Alexa Fluor 700, CD62L - BV510, CD66b - PerCP-Cy5.5, CD49d - BB515, CD16 - V450, and CD15 -RPE as well as the intracellular activation marker p38 MAPK - pT180/pY182 - RPE. Live dead cells were also measured using APC -LIVE/DEAD™ staining (ThermoFisher Scientific). The median fluorescence intensity for each marker was determined using the LSR Fortessa 4-laser flow cytometer (BD Biosciences).

### Statistical Analyses

Non-parametric descriptive and analytical statistics were applied in this study using GraphPad Prism software (San Diego, CA). The Wilcoxon signed rank test, Mann Whitney test, Pearson and Spearman ranked test correlation analyses were utilized as appropriate and *p* < 0.05 were considered statistically significant. The next generation RNA sequencing data was analyzed using the R package DESeq2 with Wald test. Here, the *p* values were adjusted for multiple testing using the Benjamini-Hochberg method for group comparisons, as previously described (19).

## Results

### Increased IL-26 Concentrations in Lower Airway Samples From Patients With Bacterial Pneumonia

**Figure 1:**
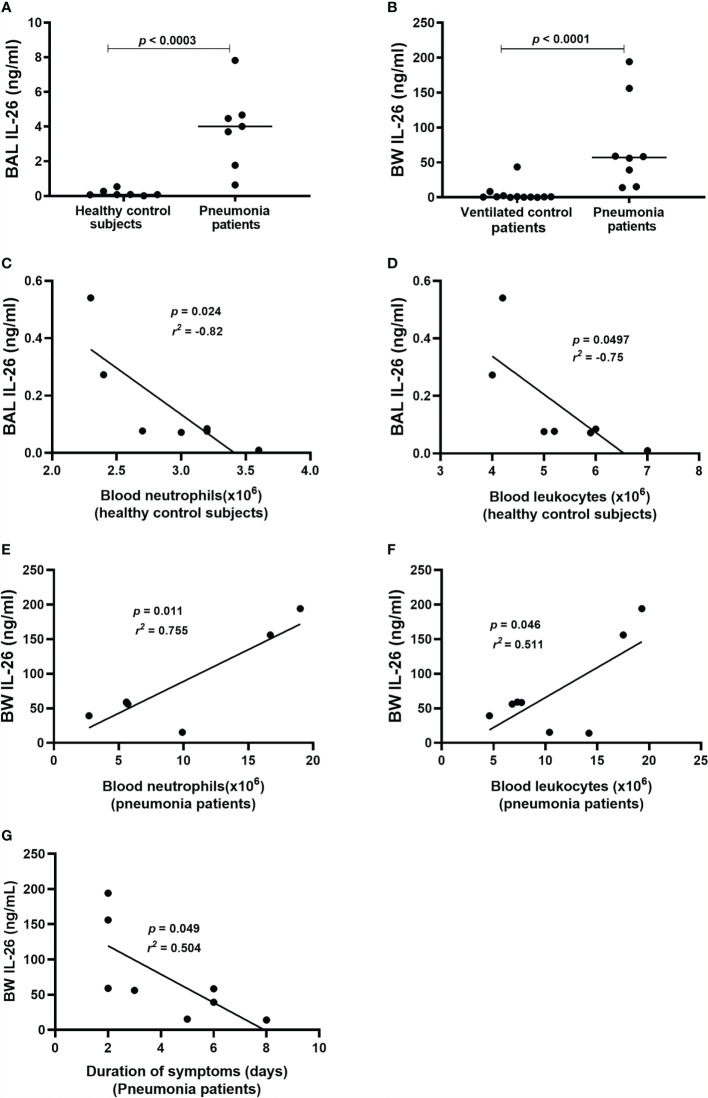
Extracellular IL-26 protein concentrations in lower airway samples during pneumonia. IL-26 protein concentrations were quantified in cell-free bronchoalveolar lavage (BAL) or bronchial wash (BW) fluid, using ELISA. Data sets are shown in panels as follows: **(A)** Concentrations of IL-26 in BAL samples from pneumonia patients (*n*=7) compared to control subjects (*n*=7); **(B)** Concentrations of IL-26 in BW samples from pneumonia patients (*n*=8) compared to control patients (*n*=12); **(C)** Concentrations of IL-26 protein in BAL samples in relation to the neutrophil concentrations in blood samples from control subjects (*n*=7); **(D)** Concentrations of IL-26 protein in BAL samples in relation to the leukocyte concentrations in blood samples from control subjects (*n*=7); **(E)** Concentrations of IL-26 protein in BW samples in relation to neutrophil concentrations in blood samples from pneumonia patients (*n*=7); **(F)** Concentrations of IL-26 protein in BW samples in relation to the leukocyte concentrations in blood samples from pneumonia patients (*n*=8); and **(G)** Concentrations of IL-26 protein in BW samples in relation to the duration of symptoms in pneumonia patients (*n*=8). The horizontal lines in **(A, B)**, indicate medians and the *p*-values are according to Wilcoxon Signed rank test. The *p-*values for **(C–G)** are according to the Pearson correlation test. *p-*values <0.05 are considered to indicate statistical significance.

To determine whether IL-26 is involved in the immune response during bacterial pneumonia, we quantified extracellular concentrations of IL-26 in lower airway (BAL & BW) samples from patients with bacterial pneumonia. In addition, clinical control samples were analyzed. The principal clinical and laboratory characteristics of the pneumonia patients and their matching controls are presented in **Table 1**, **Table 2** showing BAL and BW, respectively. We found a substantial increase in the average extracellular protein concentration of IL-26 in the BAL (**Figure 1A**) and BW samples (**Figure 1B**) from pneumonia patients compared to corresponding control samples (66-fold in the BAL & 52-fold in the BW samples). The BAL samples were harvested from pneumonia patients with ventilation (*n*=7), whereas the BAL control samples were harvested in non-ventilated healthy volunteers (*n*=7). To ascertain that our methodological approach did not introduce a technical confounder, we determined that there was no systematic difference in the median protein concentration of IL-26 protein in the BAL samples for these patients with or without ventilation (**Supplementary Figure 1A**). Moreover, all the examined BW samples were consistently harvested from ventilated pneumonia patients (*n*=8; except for one pneumonia patient that was not ventilated), and from ventilated control patients (*n*=12).

Furthermore, we observed a strong negative correlation between IL-26 concentrations in BAL samples and blood neutrophil concentrations in control subjects (**Figure 1C**), and a corresponding moderate correlation with blood leukocyte concentrations (**Figure 1D**). In contrast, there was a trend towards a positive correlation between IL-26 concentrations in BAL samples and blood neutrophil and leukocyte concentrations in the pneumonia patients (**Supplementary Figures 1B, C**). Moreover, we observed a strong positive correlation between IL-26 concentrations in BW samples and blood neutrophil concentrations in the pneumonia patients (**Figure 1E**), and a corresponding moderate correlation with blood leukocyte concentrations (**Figure 1F**). On the contrary, there was a trend towards a negative corresponding correlation in the control patients (**Supplementary Figures 1D, E**). In addition, we found that the IL-26 concentrations in BW samples correlated in a negative manner with the duration of symptoms in pneumonia patients (**Figure 1G**) and there was a trend in the same direction for the IL-26 concentrations in BAL samples (**Supplementary Figure 1F**). Data on the neutrophil and leukocyte concentrations in the BAL or BW samples were not available for clinical/technical reasons.

### Release of IL-26 From A549 Cells and Neutrophils After Bacterial Exposure

**Figure 2:**
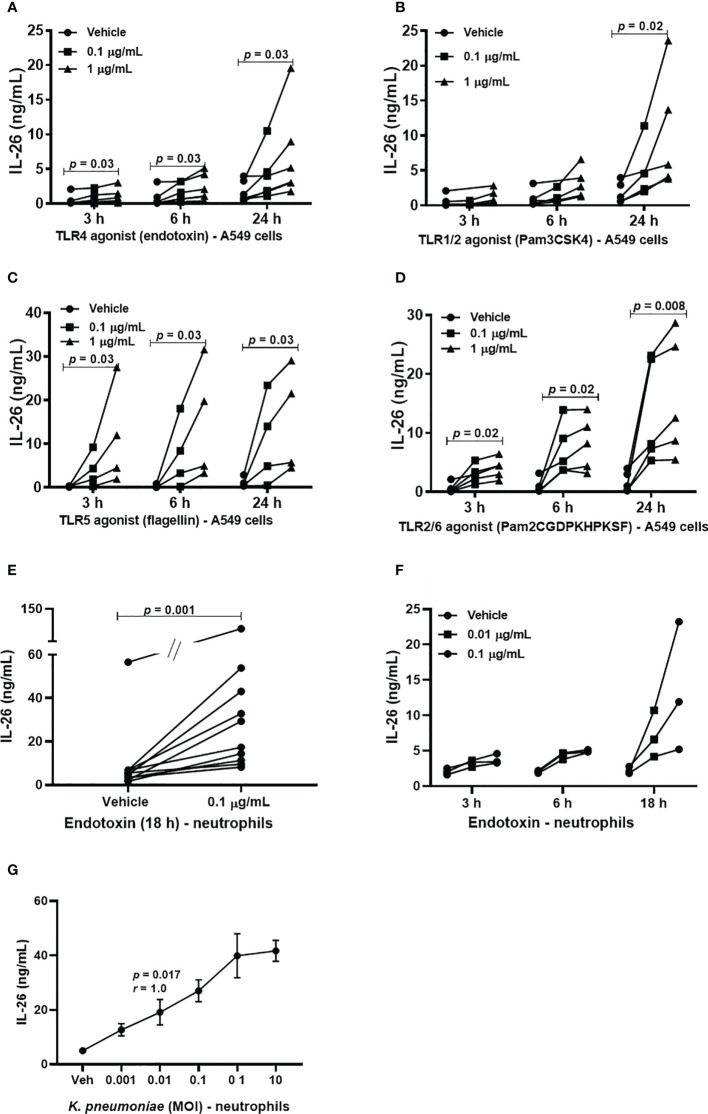
Extracellular IL-26 protein concentrations in A549 cells and neutrophils with and without exposure to bacterial stimuli. A human model of type II alveolar cells (A549 cells) was exposed to toll-like receptor (TLR) ligands, and neutrophils to endotoxin. or *K. pneumoniae*. Protein concentrations were then quantified in the cell-free conditioned media using ELISA. Panels **(A–D)** represents IL-26 concentrations in the conditioned media from A549 cells after stimulation with; **(A)** TLR1/2 ligand (*n*=5); **(B)** TLR4 ligand (*n*=5); **(C)** TLR5 ligand (*n*=4); and **(D)** TLR6 ligand (*n*=5). Panels **(E, F)** represents IL-26 concentrations in neutrophils after to: **(E)** Endotoxin for 18 h (*n*=10); and **(F)** endotoxin at different time points (*n*=3). **(G)** Panel **(G)** represents IL-26 protein concentrations after exposure (3 hours) to live *K. pneumoniae* (*n*=4). The *p-values* for panels **(A–D, F)** are according to Mann Whitney U-test. The graph in panels **(G)** is presented with standard error of the means (SEM) and computed according to the Spearman rank correlation test. *p-*values <0.05 are considered to indicate statistical significance.

Although alveolar epithelial cells are considered structural cells, these cells are also known to play a major role in inflammatory conditions in the lung parenchyma, and in innate immune responses against bacterial in the lungs (20, 21). Until now, it has not been known whether type II alveolar epithelial cells or transformed models of these cells produce IL-26 and the same is the case for neutrophils, a critical effector cell in the innate immune response against bacteria. Given these facts, we cultured A549 cells and stimulated these model cells with TLR1/2, 4, 5 and 6 ligands. Moreover, human blood neutrophils were utilized as a model of airway neutrophils for practical reasons. These neutrophils were then exposed to the TLR4 ligand LPS (endotoxin) derived from E coli. In the cell-free conditioned media from the A549 cells, we detected increased extracellular concentration of IL-26 in response to stimulation with TLR1/2, (**Figure 2A**), TLR4 (**Figure 2B**), TLR5 (**Figure 2C**) and TLR6 ligands (**Figure 2D**) in a very reproducible manner. A similarly reproducible increase in IL-26 concentration was detected in cell-free conditioned media from neutrophils in response to stimulation with endotoxin (**Figure 2E**). Notably, the respective concentrations of the stimuli are connected by a single line (**Figures 2A–D, F, G**). Furthermore, we found that the concentration of IL-26 in media from unexposed A549 cells **(** **Figures 2A–D**) and neutrophils (**Figure 2F**) tended to be stable over time, whereas the exposure to the TLR ligands tended to cause a time-dependent increase in IL-26 concentrations in the conditioned media. In contrast, the various TLR ligands caused no substantial alterations in mRNA levels for IL-26 (**Supplementary Figures 2A–D**). Importantly, exposure of neutrophils to live *K. pneumoniae* increased the median concentration of extracellular IL-26 in media in a concentration-dependent (i.e., MOI-dependent) manner after 3 h of culture (**Figure 2G**). In analogy, exposure to *K. pneumoniae* increased the median extracellular concentration of IL-8, myeloperoxidase (MPO) and neutrophil elastase (NE), (**Supplementary Figures 3A–C**).

### Stimulation With IL-26 Inhibits the Endogenous Production of Neutrophil Elastase and Myeloperoxidase in Neutrophils

**Figure 3:**
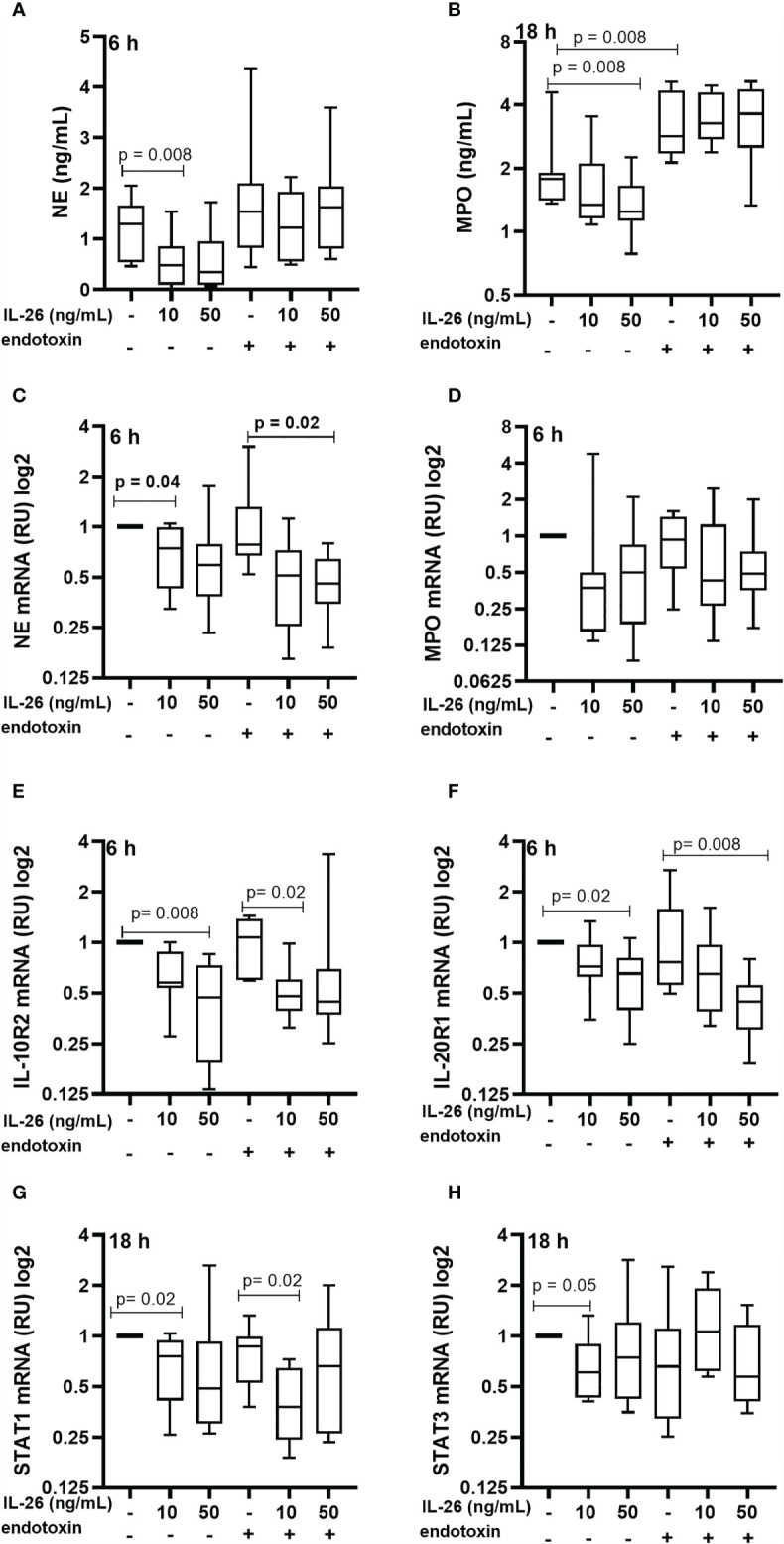
Effects of IL-26 on the activation state of neutrophils exposed to endotoxin. Human blood neutrophils were stimulated with rhIL-26 (10 and 50 ng/mL) with and without additional stimulation with endotoxin for 6 and 18 hours, respectively. The concentrations of neutrophil elastase (NE) and myeloperoxidase (MPO) were determined using ELISA. The mRNA levels of NE, MPO, IL-10R2, IL-20R1, STAT1 and STAT3 were determined using real time PCR. Data sets are shown in panels as follows: **(A)** NE protein concentrations (*n*=8); **(B)** MPO protein concentrations (*n*=8); **(C)** mRNA levels of NE (*n*=8); **(D)** mRNA levels of MPO (*n*=8); **(E)** mRNA levels of IL-10R2 (*n*=8); **(F)** mRNA levels of IL-20R1 (*n*=8); **(G)** mRNA levels of STAT1 (*n*=8); and **(H)** mRNA levels of STAT3 (*n*=8). The results are presented as median with range and the *p-values* are according to Wilcoxon Signed-rank test. *p-*values <0.05 are considered to indicate statistical significance.

To characterize the effect of IL-26 on human blood neutrophils were stimulated with rhIL-26 protein for 6 and 18 h, with and without the simultaneous exposure to endotoxin (100 ng/mL). Notably, in this case the stimulation with rhIL-26 decreased the median extracellular protein concentration of NE at 6 h (**Figure 3A**) and MPO at 18 h (**Figure 3B**). However, there was no corresponding impact on the respective median concentration of these proteins during simultaneous exposure to endotoxin. Moreover, rhIL-26 exerted no clear effects on the median extracellular NE protein concentration at 18 h and MPO at 6 h (data not shown). In analogy, stimulation with rhIL-26 decreased the (median) level of mRNA for NE, with and without endotoxin exposure (**Figure 3A**) at 6 h; with a similar trend observed for the level of mRNA for MPO (**Figure 3B**). Furthermore, rhIL-26 decreased the respective mRNA level for IL-10R2 (**Figure 3E**) and IL-20R1 (**Figure 3F**) at 6 h, regardless of simultaneous endotoxin exposure. Moreover, at 18 h stimulation with rhIL-26 decreased the STAT1 mRNA level **(** **Figure 3G**), in the absence or presence of endotoxin. We also observed a similar trend for the (median) STAT3 mRNA levels when incubated with rhIL-26 for 18 h, but only in neutrophils unexposed to endotoxin (**Figure 3H**).

### Stimulation With IL-26 Inhibits Neutrophil Activation Caused by the Exposure to *K. pneumoniae*

**Figure 4:**
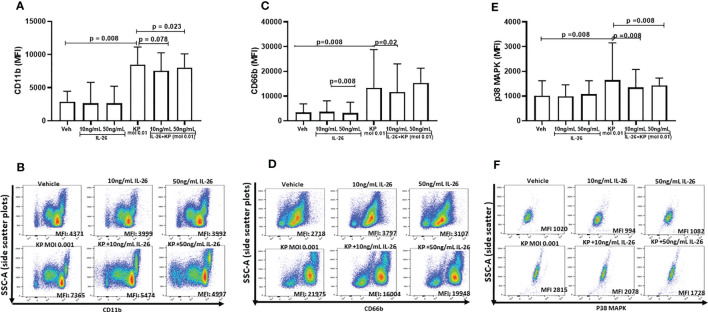
Effects of IL-26 on the activation state of neutrophils exposed to *Klebsiella pneumoniae*. Human blood neutrophils were exposed to live *Klebsiella pneumoniae* (multiplicity infection (MOI); 0.01) with and without additional stimulation by rhIL-26 (10 and 50 ng/mL) for 3 hours. The expression of CD11b, CD66b and p38 MAPK was assessed using flow cytometry and their median florescent intensity (MFI) determined. Data sets are shown in panels as follows: **(A)** MFI for CD11b expression for all subjects (*n*=8); **(B)** Representative scatter plots for CD11b expression; **(C)** MFI for CD66b for all subjects (*n*=8); **(D)** Representative scatter plots for CD66b expression; **(E)** MFI for p38 MAPK expression for all subjects (*n*=9); **(F)** Representative scatter plots for p38 MAPK expression. The results in panels **(A, C, E)** are presented as median with range and the *p-values* are according to Wilcoxon Signed-rank test. *p-*values <0.05 are considered to indicate statistical significance.

To further dissect the effects of IL-26 on neutrophil activity, human blood neutrophils were exposed to live *K. pneumoniae* (MOI 0.01), with and without stimulation with rhIL-26. After 3 h, the surface expression of CD11b and CD66b was quantified in addition to the intracellular protein level of phosphorylated p38 MAP kinase. The initial, preliminary experiments indicated that 3 h was the optimal time point for effects of rhIL-26. Utilizing this time for stimulation, we observed that rhIL-26 (50 ng/mL) inhibited the (median) surface expression of CD11b in neutrophils exposed to *K. pneumoniae* (**Figure 4A**). In contrast, rhIL-26 did not markedly alter this surface protein expression in unexposed neutrophils (**Figure 4B**). Likewise, rhIL-26 (10 ng/mL) inhibited the (median) surface expression of CD66b in neutrophils exposed to *K. pneumoniae* (**Figures 4C, D**), whereas rhIL-26 did not markedly alter CD66b expression in in neutrophils not exposed to this bacterial species. Furthermore, rhIL-26 (10 or 50 ng/mL) inhibited the (median) phosphorylation of p38 MAPK caused by *K. pneumoniae* (**Figures 4E, F**), whereas rhIL-26 did not markedly alter this phosphorylation in unexposed neutrophils. Notably, stimulation with rhIL-26 did not significantly alter the (median) surface expression of neither CD62L nor CD16 (**Supplementary Figures 4A–D**), nor that of CD49d or CD15 (**Supplementary Figures 5A–D**) in neutrophils with or without simultaneous exposure of *K. pneumoniae*.

### Monocyte-Derived Macrophages Release IL-26 as Well as IL-6, IL-8 and IL-10 After Exposure to Gram-Negative Bacteria

**Figure 5:**
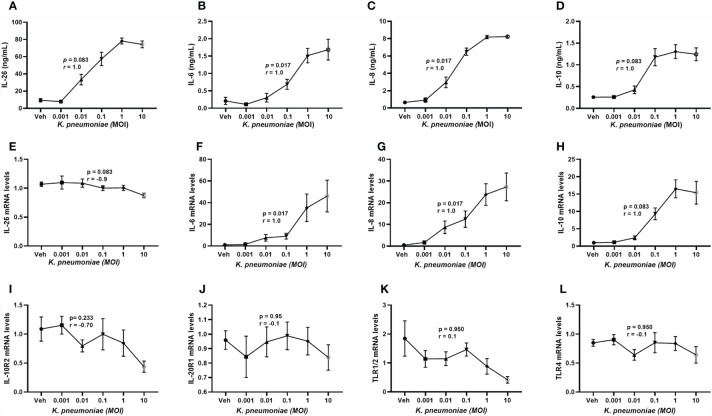
Extracellular IL-26, IL-6, IL-8, and IL-10 protein concentrations and levels of matching mRNA in macrophages with and without exposure to *K. pneumoniae*. Monocyte-derived macrophages were exposed to *K. pneumoniae* (3 hours) at different multiplicities of infection (MOI; 0.001, 0.01, 0.1, 1 and 10 and vehicle). Protein concentrations were then quantified in the cell-free conditioned media using ELISA and cellular mRNA was measured using qRT-PCR. Data sets are shown in panels as follows: **(A)** IL-26 protein concentrations (*n*=7); **(B)** IL-6 protein concentrations (*n*=7); **(C)** IL-8 protein concentrations (*n*=7); **(D)** IL-10 protein concentrations (*n* = 7); **(E)** IL-26 mRNA levels (*n*=8); **(F)** IL-6 mRNA levels (*n*=8); **(G)** IL-8 mRNA levels (*n*= 8); **(H)** IL-10 mRNA levels (*n*=8); **(I)** IL-10R2 mRNA levels (*n*=8); **(J)** IL-20R1 mRNA levels (*n*=8); **(K)** TLR1/2 mRNA levels (*n*=4); and **(L)** TLR4 mRNA levels (*n*=8). The results are presented as standard error of the means (SEM) and dose response data are according to the Spearman rank-correlation test. *p-*values <0.05 are considered to indicate statistical significance.

It is known that primary alveolar macrophages produce IL-26 in response to exposure of *E. coli* endotoxin (6, 8, 9) but the corresponding response to live *K. pneumoniae* has not yet been characterized in human macrophages or other cells of the monocyte lineage. Utilizing monocyte-derived macrophages (MDM), we found that exposure to increasing concentrations of live *K. pneumoniae* enhanced the median extracellular concentration of IL-26 in conditioned media from MDM in a concentration-dependent (ie. MOI-dependent) manner (**Figure 5A**). Notably, the exposure to *K. pneumoniae* also enhanced the release of the (median) IL-6 (**Figure 5B**), IL-8 (**Figure 5C**), and IL-10 protein concentration (**Figure 5D**). In contrast, when cells were exposed to *K. pneumoniae*, we observed no substantial effects on the (median) level of mRNA for IL-26 **(** **Figure 5E**). However, the respective level of mRNA for IL-6 (**Figure 5F**), IL-8 (**Figure 5G**), and IL-10 (**Figure 5H**) was increased by exposure to *K. pneumoniae*. In contrast, we observed no statistically significant effect on the median mRNA level for IL-10R2 (**Figure 5I**), IL-20R1 (**Figure 5J**), TLR1/2 (**Figure 5K**), or TLR4 (**Figure 5L**) that was caused by the exposure to *K. pneumoniae*. However, the (median) level of mRNA for IL-10R2 was suppressed, albeit not in a statistically significant manner.

### Stimulation With IL-26 Activates Monocyte-Derived Macrophages to Release IL-10 but Not IL-6 nor IL-8 After Exposure to *K. pneumoniae*

**Figure 6:**
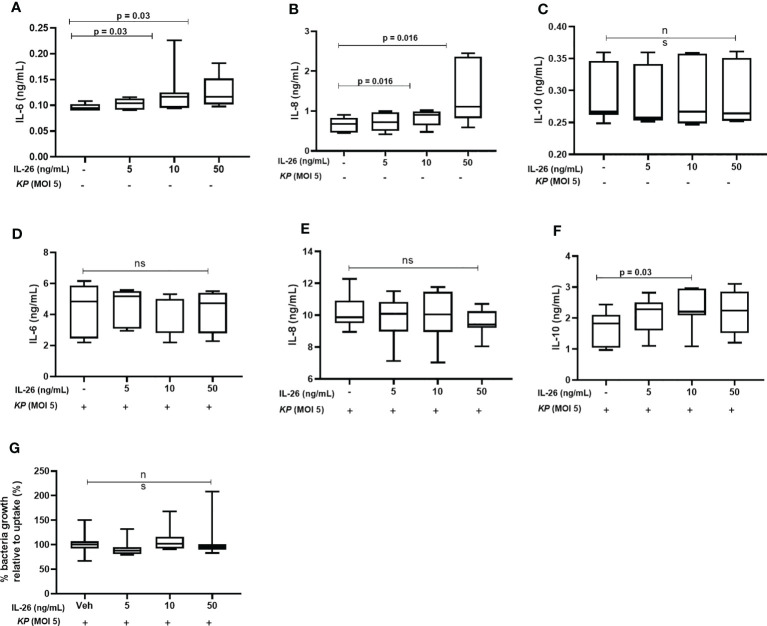
Effects of exogenous IL-26 on extracellular cytokine concentrations and the growth of *Klebsiella pneumoniae* in macrophages. Human monocyte-derived macrophages were treated with different concentrations of rhIL-26 and vehicle (Veh) as control, for 18 hours. Cytokine protein concentrations were measured in the cell-free conditioned media using ELISA. Data sets are shown in panels **(A–C)** as follows: **(A)** IL-6 concentrations (*n*=7); **(B)** IL-8 concentrations (*n*=7); **(C)** IL-10 concentrations (*n*=7). The IL-26-treated macrophages were subsequently exposed to *K. pneumoniae* (MOI 5) washed and cultured in fresh culture media (10% FCS in RPMI) for 4 hours (37°C). The cytokine protein concentrations were again measured in the cell-free conditioned media using ELISA. Additional data sets are shown in panels **(D–F)** as follows: **(D)** IL-6 concentrations (*n*=7); **(E)** IL-8 concentrations (*n*=7); and **(F)** IL-10 concentrations (*n*=7). Extracellular bacteria were subsequently killed (gentamycin, 25ug/mL for 30 minutes at 37°C) and washed off the cells. The cells were then lysed, and the lysate plated on blood agar overnight. Panel **(G)** shows the percentage of bacteria growth relative to uptake 6 h (n=6). The data are presented as median with range and the *p-values* are according to Wilcoxon signed-rank test. *p-*values <0.05 are considered to indicate statistical significance.

It has previously been shown that stimulation by IL-26 enhances IL-6 and IL-8 mRNA levels in alveolar macrophages and unsorted BAL cells (8, 9). Here, we investigated whether IL-26 stimulates the release of IL-10, IL-6, and IL-8 in MDM. Interestingly, while stimulation by rhIL-26 enhanced the (median) extracellular protein concentration of both IL-6 (**Figure 6A**) and IL-8 (**Figure 6B**) in conditioned media, this stimulation caused no clear effect on the corresponding concentration of IL-10 (**Figure 6C**). In contrast, we observed no increase in the (median) extracellular concentration of either IL-6 (**Figure 6D**) or IL-8 (**Figure 6E**) when the rhIL-26-primed MDM were subsequently exposed to live *K. pneumoniae* (MOI 5). Interestingly, there was an increase in the (median) IL-10 concentration (**Figure 6F**) in conditioned medium when the rhIL-26-primed MDM were subsequently exposed to live *K. pneumoniae*. Moreover, when the extracellular bacteria were removed by extensive washing steps (treatment with gentamicin (25 μg/mL for 30 min at 37°C) and washed (3 x) with PBS), followed by lysis and culture on blood agar, we found no differences in the growth of *K. pneumoniae* for the primed and unprimed MDM (**Figure 6G**).

### Stimulation With IL-26 Primes Human Lung Tissue to Increased Expression of Both Pro- and Anti-Inflammatory Cytokines

**Figure 7:**
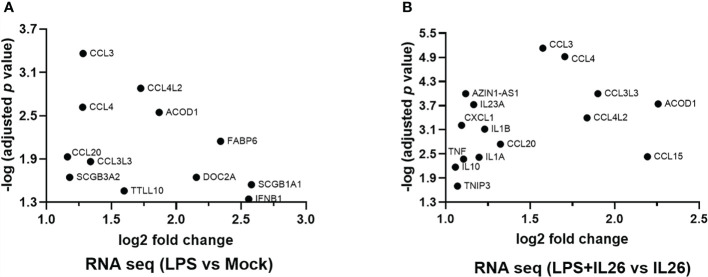
Effects of exogenous IL-26 on gene transcription in lung tissue. An average of 30 mg of lung tissue from human patients (n=8) was cultured in cell medium (see methods) and stimulated with rhIL-26 (100 ng/mL), with or without the exposure to endotoxin (100 ng/mL) overnight. RNA was extracted and sequenced according to the Illumina^®^ technology (see methods). The data presented was filtered such that only the genes displaying 2 log-fold change in transcription as well as -1.3 log-fold of *p-*value were considered. Data sets are shown in panels **(A, B)** as follows: **(A)** Gene transcripts in response to endotoxin exposure; and **(B)** Gene transcripts in response to endotoxin after priming by rhIL-26.

To further explore the “priming” effects of IL-26 on immune signaling in response to bacterial exposure, we exposed fresh human lung tissue *ex vivo* to endotoxin from Gram-negative bacteria, with and without simultaneous stimulation with rhIL-26. Next generation RNA sequencing (NGS RNA) analyses were performed on the tissue samples, and we restricted our analyses below to induced changes in mRNA levels that displayed at least a 2-log-fold magnitude and an adjusted *p-*value <0.05 (equal to or exceeding -log 1.3). In doing so, we observed that the exposure to endotoxin alone increased the (median) level of mRNA for the chemokines CCL3, CCL4L2, CCL4, CCL20, and CCL3L3 (**Figure 7A**). Moreover, simultaneous stimulation with rhIL-26 caused a further enhancement of the levels of mRNA for the above-mentioned chemokines, plus an additional enhancement of CXCL1 and CCL15 (**Figure 7B**). Furthermore, we found that the simultaneous stimulation with rhIL-26 also increased the mRNA level for the anti-inflammatory compounds IL-10, TNFIP3 and aconitate decarboxylase 1 (ACOD1). In addition, this simultaneous stimulation increased the (median) mRNA level for the pro-inflammatory cytokines IL-23A, IL-1β, IL1A, and TNF-α in response to endotoxin (**Figure 7B**). Notably, we observed the increase in (median) mRNA level for IFN-β and host-defense-unrelated mediators such as FABP6, SCGB3A2, SCGB3A1 and DOC2A, after exposure to endotoxin alone. However, we observed no alteration in the mRNA levels of these after the simultaneous co-stimulation with rhIL-26 (**Figure 7B**).

### Endogenous IL-26 Regulates Cytokine Release and Inhibits Bacterial Growth in Monocyte-Derived Macrophages

**Figure 8:**
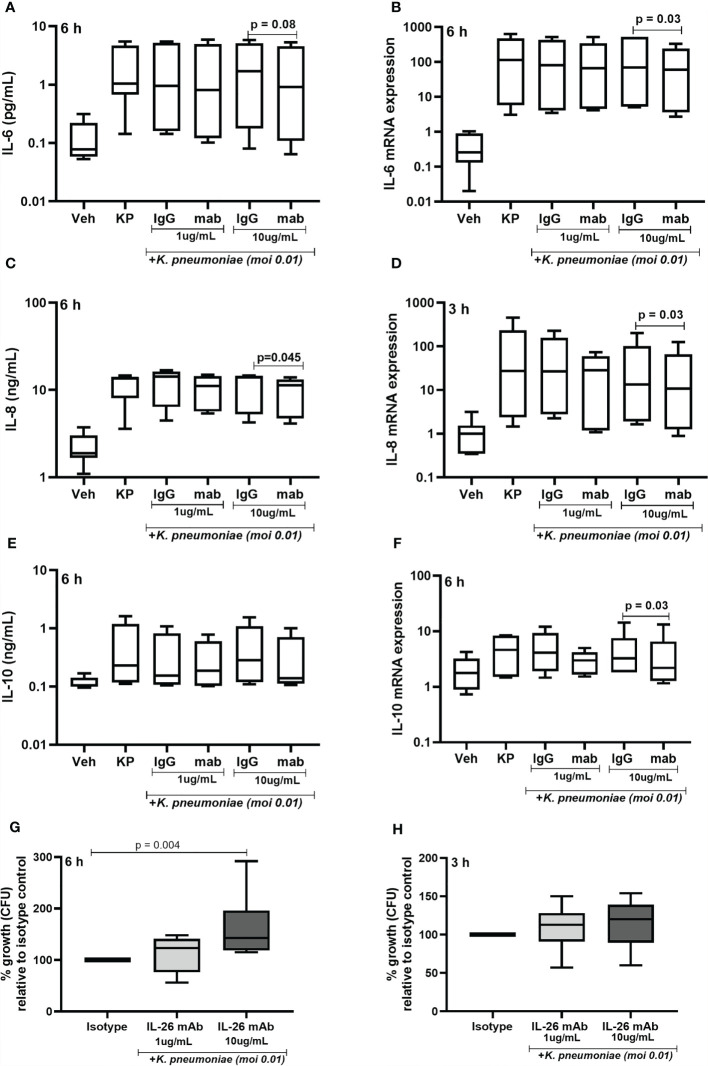
Effects of endogenous IL-26 on extracellular cytokine concentrations and the growth of *Klebsiella pneumoniae* in macrophages. Human monocyte-derived macrophages were stimulated simultaneously with *Klebsiella pneumoniae* (MOI; 0.01), with or without a neutralizing anti-IL-26 monoclonal antibody or the IgG1 isotype control (3 and 6 hours respectively). Cytokine protein concentrations were quantified (ELISA) in the cell-free conditioned media and cellular mRNA (qRT-PCR). Data sets are shown in panels as follows: **(A)** IL-6 concentrations after 6 hours (*n*=7); **(B)** IL-6 mRNA levels after 6 hours (*n*=6); **(C)** IL-8 concentrations after 6 hours (*n*=6); **(D)** IL-8 mRNA after 3 hours (*n*=6); **(E)** IL-10 concentration after 6 hours (*n*=7); and **(F)** IL-10 mRNA after 6 hours. Extracellular bacteria were killed (gentamycin, 25ug/mL for 30 minutes at 37°C) and washed off the cells. The cells were then lysed, and the lysate plated on blood agar overnight. Bacteria colony forming units (CFU) were then counted manually and the number of bacteria was calculated. Data shown in panels as follows: **(G)** Percentage of bacteria growth relative to the isotype control after 6 hours (*n*=6); and **(H)** corresponding data after 3 h (*n*=6). The results are presented as median with range, and the *p-values* are according to Wilcoxon signed-rank test. *p-*values <0.05 are considered to indicate statistical significance.

Given that exposure to live *K. pneumoniae* increases the release of IL-26 protein in MDM, we investigated whether the endogenous IL-26 regulates the release of IL-6, IL-8, and IL-10 protein. Importantly, we also investigated whether endogenous IL-26 released by MDM is involved in the control of bacterial growth. To do this, endogenous IL-26 was neutralized using a specific mAb, while simultaneously exposing MDM to *K. pneumoniae* (MOI 0.01). Notably, we observed that the neutralization of endogenous IL-26 clearly decreased the (median) level of extracellular protein in conditioned media and of the level intracellular mRNA for IL-6 **(** **Figures 8A, B**), IL-8 (**Figures 8C, D**) and IL-10 (**Figures 8E, F**). We thereafter removed extracellular or cell-bound bacteria by extensive washing steps (treatment with gentamicin (25 μg/mL for 30 min at 37°C) and washed (3 x) with PBS), followed by lysis and culture on blood agar. We then observed that the treatment with the neutralizing IL-26 antibody caused a substantial increase in the CFU for *K. pneumoniae* at 6 h (**Figure 7G**). In support of this, we found a matching trend at 3 h, although the effect at this time point was not statistically significant (**Figure 8H**).

## Discussion

This study on IL-26 in bacterial lung infection presents seven fundamental observations. First, we observed that the median extracellular IL-26 concentration was markedly increased in lower airway samples from patients with pneumonia. Second, we observed that the extracellular IL-26 concentrations in lower airway samples correlated in a positive manner with leukocyte and neutrophil concentrations in peripheral blood from patients with pneumonia. Third, we observed that the exposure to PAMPs from Gram-negative and Gram-positive bacteria increased extracellular concentrations of endogenous IL-26 protein in a model of human type II alveolar epithelial cells. Fourth, we observed that live *K. pneumoniae*, just like endotoxin from Gram-negative bacteria, enhanced extracellular IL-26 concentrations in conditioned media from human blood neutrophils. Moreover, in this neutrophil model, exogenous IL-26 inhibited the inherent release of NE and MPO and corresponding mRNA levels, including IL-10R2, IL-20R1, and STAT1. In addition, exogenous IL-26 inhibited the expression of the neutrophil activity markers CD11b, CD66b as well as the intracellular p38 MAPK phosphorylation that is caused by *K. pneumoniae*. Fifth, we observed that live *K. pneumoniae* enhanced extracellular concentrations of IL-26 protein in conditioned media from human MDMs, and that endogenous IL-26 from these macrophages inhibited the growth of the same Gram-negative bacterial species. Sixth, we observed that priming of macrophages with exogenous IL-26 increased extracellular IL-10 concentrations in conditioned media after exposure to *K. pneumoniae.* Seventh, we observed that priming of human lung tissue with exogenous IL-26 further enhanced the endotoxin-induced increase in mRNA for several critical immunoregulating cytokines, including IL-23 and IL-10. Taken together, our findings demonstrate that IL-26 is involved in the innate immune response during bacterial pneumonia in humans, that this cytokine may both enhance and limit this immune response, and that endogenous IL-26 triggered by a Gram-negative bacterial species contributes to the killing of the same bacteria. The investigated patients with pneumonia displayed substantially increased IL-26 concentrations in two types of lower airway samples, including BAL and BW samples. Moreover, IL-26 in the BW samples from patients with pneumonia displayed a positive correlation with the leukocyte and neutrophil concentrations in blood, and a similar trend was observed in the BAL samples. However, in contrast to the pneumonia patients, we found that IL-26 concentrations in BAL correlated in a negative manner with leukocyte and neutrophil concentrations in the blood of control subjects, and the same trend was observed in BW samples. The herein described “opposing” associations between local IL-26 and leukocyte and neutrophil concentrations in the blood from patients with bacterial pneumonia and from control subjects, respectively, represent truly novel findings. The findings are compatible with the hypothesis that, during a bacterial infection in human lungs, local IL-26 increases the mobilization of leukocytes and neutrophils in the blood to enhance recruitment into the target organ, whereas under normal physiological conditions, local IL-26 curtails the mobilization of leukocytes and neutrophils in blood, to prevent any inherent recruitment and subsequent accumulation in the lungs. These *in vivo* findings are in line with mechanistic evidence obtained *in vitro*, whereby rhIL-26 increases the migration of neutrophils (chemotaxis) in the presence of disease-induced inflammatory signals (IL-8 or fMLP) whereas this cytokine inhibits the inherent migration of neutrophils (chemokinesis) in the absence of these signals (9). Given that there is overwhelming evidence showing that there is accumulation of leukocytes and neutrophils in human lungs during pneumonia (22, 23), our finding is suggestive of IL-26 being involved in the mechanisms behind this accumulation. In contrast, IL-26 may play the opposite role during normal conditions, although it remains to be addressed whether and how this dualistic impact relates to receptor biology. Finally, it is worth noticing that the utilized BW samples reflect the large airways, and BAL reflect the small airways. This means that the relationship between local IL-26 and blood neutrophils may vary for anatomical reasons as well, although not in such a dramatic manner as for patients and control subjects.

Our experimental results present evidence that pathogenically relevant bacterial stimuli can cause the release of IL-26 from cells being locally present during bacterial lung infection. For example, in a human model of type II alveolar epithelial cells (21) PAMPs from both Gram-negative and Gram-positive bacteria *in vitro*, including endotoxin (Gram-negative bacteria), and lipopeptides and flagellin (Gram-negative and Gram-positive bacteria), as well as lipopeptides derived from *Mycoplasma*, all enhanced the release of IL-26 into the extracellular compartment. These data on A549 cells provides for future studies on the functional effects of IL-26 on primary alveolar epithelial cells given that alveolar epithelial cells reside in the most peripheral airways/the parenchyma of the lungs, an area where severe pathological alterations occur during bacterial infection. Along the same lines, in our model of airway neutrophils and macrophages, respectively, live *K. pneumoniae* caused reproducible release of IL-26 protein into the extracellular space. Notably, given that these innate effector cells are recruited in very high numbers into the site of infection during bacterial pneumonia (22–24), even a modest cytokine response for each individual effector cell is collectively likely to make a functional impact *in vivo*. Thus, these novel findings on neutrophils and A549 cells, as well as the confirmatory finding on MDM, forward locally accumulated neutrophils, macrophages, and alveolar type II cells as potentially important sources of extracellular IL-26 protein in bacterial pneumonia. However, we found no corresponding effect on the level of IL-26 mRNA in alveolar epithelial cells, macrophages, or neutrophils in response to the bacterial stimuli. This consistent finding indicates that there is relatively rapid release of prestored IL-26 rather than enhanced gene transcription during bacterial infection in human lungs. This interpretation is supported by the fact that type II alveolar epithelial cells, macrophages, and neutrophils released IL-26 as early as 3 h after the exposure to bacterial stimuli. Indeed, there is further evidence supporting this hypothesis in the literature showing that alveolar macrophages, T cells (6, 8, 9), bronchial epithelial cells (10), and lung fibroblasts (11) isolated from healthy human lungs display prestored as well as inherent release of IL-26 protein (6). Moreover, it is noteworthy that the protein concentrations of IL-26 in BW samples decreased with the duration of symptoms, an observation that is compatible with this cytokine reflecting early and critical events during bacterial lung infection. The experimental results also provided us with evidence that IL-26 modulates not only the local accumulation of neutrophils, but also their state of activation.

We observed that stimulation with rhIL-26 protein alone decreased the respective constitutive release of NE and MPO protein in neutrophils. Thus, we obtained evidence that IL-26 inhibits the release of NE, an entirely novel observation confirming that IL-26 may modulate the inherent release of a potent secretagogue in neutrophils, just as it does with MPO (9). Moreover, we obtained evidence that rhIL-26 inhibited the transcription of NE and MPO as well as IL-10R2, IL-20R1, and STAT1 in neutrophils, all being entirely novel observations. Here, it is noteworthy that IL-26 is already known to inhibit the phosphorylation of STAT3 in neutrophils (9), suggesting that IL-26 inhibits STAT3 both at the gene level as well as its phosphorylation. Furthermore, it remains to be shown whether IL-26 can modulate NE or MPO in neutrophils exposed to some bacterial stimulus other than endotoxin. Moreover, we observed that stimulation with rhIL-26 decreased the expression of CD11b and CD66b, two archetype surface proteins indicating the activation state of human neutrophils (25). Likewise, we found that rhIL-26 decreased the induced phosphorylation of p38 MAPK that was caused by the exposure to *K. pneumoniae.* These findings argue in favor of a negative feedback mechanism by which IL-26, through self-inhibition of the gene expression for its own receptors (IL-10R2, IL-20R1), its intracellular signaling molecules STAT1 as well as the phosphorylation of STAT3 (9) leads to a decreased production of NE and MPO, as well as lower activation state of neutrophils. Indeed, these findings are also suggestive that IL-26 exerts a dualistic immunomodulatory effect on neutrophil mobilization, fully along the lines of what is known about its inhibition of neutrophil chemokinesis, and potentiation of chemotaxis to a bacterial compound (fMLP) and a chemokine (IL-8) (9). Notably, although the inhibition of NE and MPO may be beneficial in limiting tissue destruction during the inflammatory process in the lungs during bacterial pneumonia, this may also be a limiting factor given that NE and MPO also mediate bacterial killing (26). Thus, a further characterization of how IL-26 stimulates the accumulation of neutrophils in the airways (12), while at the same time exerting immunomodulatory effects on the activation state and release of antibacterial compounds such as NE and MPO, may prove useful for identifying novel therapeutic targets for modulation of neutrophil mobilization during bacterial lung infection.

Utilizing a specific and neutralizing antibody targeting IL-26 in a human model of lung macrophages exposed to live *K. pneumoniae*, we found that inhibition of the endogenous IL-26 clearly increased the growth of this Gram-negative bacterial pathogen. Of note, alveolar macrophages are known to produce IL-26 but the effects are unknown (8, 9). Thus, we think that this is an important finding, arguing that endogenous IL-26 protein from lung macrophages in response to bacteria, may contribute to the direct killing of the bacteria in the lungs. Indeed, this evidence adds to previously published experimental evidence that exogenous IL-26 protein exerts bactericidal effects on bacteria by inducing membrane pore formation and subsequent ion leakage in the Gram-negative species *P. aeruginosa* (13). However, our prior priming with exogenous IL-26 protein before exposure to *K. pneumoniae* did not exert any detectable effect on the growth of the bacteria in the utilized macrophage model, and this is compatible with supra-maximal concentrations of IL-26 already being present due to endogenous production. In support of our observation, a similar effect of a neutralizing anti-IL-26 antibody was previously demonstrated in human Th17 cells cultured *in vitro*, in which this neutralizing antibody enhanced the growth of *P. aeruginosa*, another Gram-negative bacterial species that bears pathogenic relevance for bacterial lung infection (13). Importantly, these findings may represent fundamental mechanisms in host defense against bacteria, given that resident macrophages are extremely abundant and constitute a critical immune barrier in the lungs as well as in other human organs. Furthermore, we found that neutralization of endogenous IL-26 in the human macrophage model caused a modest decrease in the levels of mRNA and/or protein concentrations for IL-6, IL-8, and IL-10. To what extent these modulatory effects are important for the bacterial killing in macrophages or for the optimization of the innate immune response in other ways, remains to be elucidated. Either way, this finding supports our previous studies showing that IL-26 alters the release of pro-inflammatory cytokines in human airways (8, 9, 27).

In the current study, we also addressed the priming effects of IL-26 in airway macrophages and in lung tissue from humans. Thus, in MDM’s cultured *in vitro*, our model of airway macrophages, we found that whereas stimulation by rhIL-26 caused the release of IL-6 and IL-8 protein, this cytokine did not cause any corresponding release of IL-10. However, when the MDM’s were primed with exogenous IL-26, the exposure to live *K. pneumoniae* triggered increased release of IL-10, but not of IL-6 and IL-8. This finding argues that indeed, IL-26 bears the capacity to skew macrophage towards an anti-inflammatory response after exposure to Gram-negative bacteria, a response that may serve to limit the entire innate immune response over time. Moreover, in the lung tissue cultured *ex vivo*, we observed that the priming with rhIL-26 further enhanced the endotoxin-induced increase in mRNA for the archetype anti-inflammatory cytokine IL-10, as well as for the anti-inflammatory molecules TNIP3 and ACOD1. With reference to these effects, it is of interest to note that coordinated immune signaling *via* IL-10, TNIP3 and ACOD1 is known to inhibit the generic proinflammatory transcription factor NF-κB, resulting in the suppression of the corresponding functional responses (28–31). From a hypothetical point-of-view, the effect that we observed may be a unique feature of IL-26 that positions this cytokine as modulatory factor for innate immune response, displaying a homeostatic potential that may be beneficial for ascertaining adequate but limited innate immune responses over time after activation (28, 32–34). Clearly, the herein described effects of IL-26 motivate more in-depth investigations of the specific underlying mechanisms, taking into consideration the fact that IL-26 can exert effects independent of the one receptor complex that has been described so far (6).

Finally, we found that in the lung tissue cultured *ex vivo*, priming with rhIL-26 potentiated the endotoxin-induced increase in mRNA for several cytokines that are known to promote the recruitment of leukocytes directly or indirectly. Specifically, these included CCL3, CCL4, CCL3L3, CCL4L2, CCL20, CXCL1, and CCL15, as well as pro-inflammatory cytokines IL-23, IL-1B, IL-1A, and TNFα. The increased transcription of the genes for these leukocyte recruiting factors and the proinflammatory cytokines adds more evidence that IL-26 is deeply involved in the modulation of pro-inflammatory gene transcription in the lungs, along the lines to what has been shown for NFκB, IL-1β, TNFα, IL-6 and IL-8 in alveolar macrophages (8). Moreover, these genes are known to be critically involved in the modulation of innate immune responses in the lungs (20, 35), again forwarding the possibility that IL-26 may serve as a homeostatic factor during the activation of innate immune responses in bacterial lung infection.

In summary, the results of this study demonstrate the involvement in pneumonia patients and link local IL-26 to the systemic mobilization of neutrophils in these patients but not in controls. Moreover, the results of this study indicate that macrophages and alveolar epithelial cells, resident immune barrier cells, just like extravasating blood neutrophils, innate effector cells expected to be present at the site of infection, all constitute prominent producers of IL-26 in response to bacterial stimulation. These cells thereby provide the means for substantial production of IL-26 during bacterial lung infection. Notably, this IL-26 may exert complex actions on innate immune responses to common bacterial pathogens, by modulating innate immune responses from alveolar epithelial cells, neutrophils, and macrophages. In fact, IL-26 may inhibit neutrophil activity and potentiate gene transcription for the master Th17 regulator, IL-23, and the archetype anti-inflammatory cytokine, IL-10. Finally, the results of this study suggest that endogenous IL-26 from macrophages contribute to the killing of Gram-negative bacteria. Tentatively, IL-26 may modulate neutrophil mobilization and contribute to the killing of bacteria while at the same time preventing the innate immune response from being excessive over time, thereby exerting a homeostatic effect. Given these findings, this dualistic cytokine deserves to be further evaluated with respect to diagnostic and therapeutic potential in pneumonia.

## Data Availability Statement

The raw data supporting the conclusions of this article will be made available by the authors, without undue reservation.

## Ethics Statement

The studies involving human participants were reviewed and approved by The Swedish Authority for Ethical Review (Etikprövningsmyndigheten). The patients/participants provided their written informed consent to participate in this study.

## Author Contributions

Conceptualization: KFC, MP, KR, and AL. Methodology: KFC, MP, JaS, IM, KP, and MR. Investigation: KFC, MP, KP, and MR. Visualization: KFC, MP, KP, MR, JeS, MA, and AL. Funding acquisition: AL, KR and MP. Project administration: AL, and KR. Supervision: AL, and KR. Writing – original draft and completion: KFC, and AL. Writing – review and editing: MP, KP, MR, RR, JS, MA, PB, L-OC, and KR. All authors contributed to the article and approved the submitted version.

## Funding

This study was sponsored by the: Swedish Heart-Lung Foundation (AL: #20180315; KR: #20180401); The Swedish Research Council (AL: #2016-01563; MP: #2018-06924; KR #2019-01053); Region Stockholm (AL: ALF # 2018-0088); The Swedish Society for Medical Research (MP); Anna and Edwin Berger foundation (KR) and Skåne County Council’s research and development foundation (KR). The sponsors played no role in the design of the manuscript or in the selection of its contents.

## Conflict of Interest

The authors declare that the research was conducted in the absence of any commercial or financial relationships that could be construed as a potential conflict of interest.

The editor declared a past collaboration with one of the authors, KC, at the time of review.

## Publisher’s Note

All claims expressed in this article are solely those of the authors and do not necessarily represent those of their affiliated organizations, or those of the publisher, the editors and the reviewers. Any product that may be evaluated in this article, or claim that may be made by its manufacturer, is not guaranteed or endorsed by the publisher.
