# Supplementary material for: Complex Involvement of Interleukin-26 in Bacterial Lung Infection
Source: Front Immunol. 2021 Oct 28;12:761317. doi: 10.3389/fimmu.2021.761317 (PMC8581676; doi:10.3389/fimmu.2021.761317)
Supplement: Supplementary file 1 [file DataSheet_1.pdf]

Complex Involvement of Interleukin-26 in Bacterial Lung Infection

Karlhans F. Che[1,12], Magnus Paulsson[2,3,4] Krzysztof Piersiala[5,6,] Jakob Sax[1], Ibrahim Mboob[1], Mizanur Rahman[7], Rokeya S. Rekha[8], Jesper Säfholm[9,10], Mikael Adner[9,10], Peter Bergman[8,11], Lars-Olaf Cardell[5,6], Kristian Riesbeck[2], Anders Lindén[1,12]

[1]Unit for Lung and Airway Research, Institute of Environmental Medicine, Karolinska Institutet, Stockholm, Sweden.
[2]Clinical Microbiology, Department of Translational Medicine, Faculty of Medicine, Lund University, Malmö, Sweden.
[3]Division of Infection Medicine, Department of Clinical Sciences, Faculty of Medicine, Lund University, Lund, Sweden.
[4]Department of Infectious Diseases, Skåne University Hospital, Lund, Sweden
[5]Division of ENT Diseases, Department of Clinical Sciences, Intervention and Technology, Karolinska Institutet, Stockholm, Sweden.
[6]Department of Ear Nose and Throat Diseases, Karolinska University Hospital, Stockholm, Sweden.
[7]Unit of Integrative Toxicology, Institute of Environmental Medicine (IMM), Karolinska Institutet, Stockholm, Sweden.
[8]Division of Clinical Microbiology, Department of Laboratory Medicine, Karolinska Institutet
[9]Institute of Environmental Medicine, Karolinska Institutet, Stockholm, Sweden.
[10]Centre for Allergy Research, Karolinska Institutet, Stockholm, Sweden.
[11]Immunodeficiency Unit, Department of Infectious Disease, Karolinska University Hospital, Stockholm, Sweden.
[12]Karolinska Severe Chronic Obstructive Pulmonary Disease Center, Department of Respiratory Medicine and Allergy, Karolinska University Hospital Solna, Stockholm, Sweden.

*__Corresponding author__
Professor Anders Lindén, M.D., Ph.D.,
Unit for Lung and Airway Research, Institute of Environmental Medicine,
Karolinska Institutet, PO Box 210, SE-171 77 Stockholm, Sweden
E-mail: anders.linden@ki.se, Phone: +46 (0)70 090 2286

SUPPLEMENTARY FIGURES, TABLES AND LEGENDS

Supplementary Figure 1

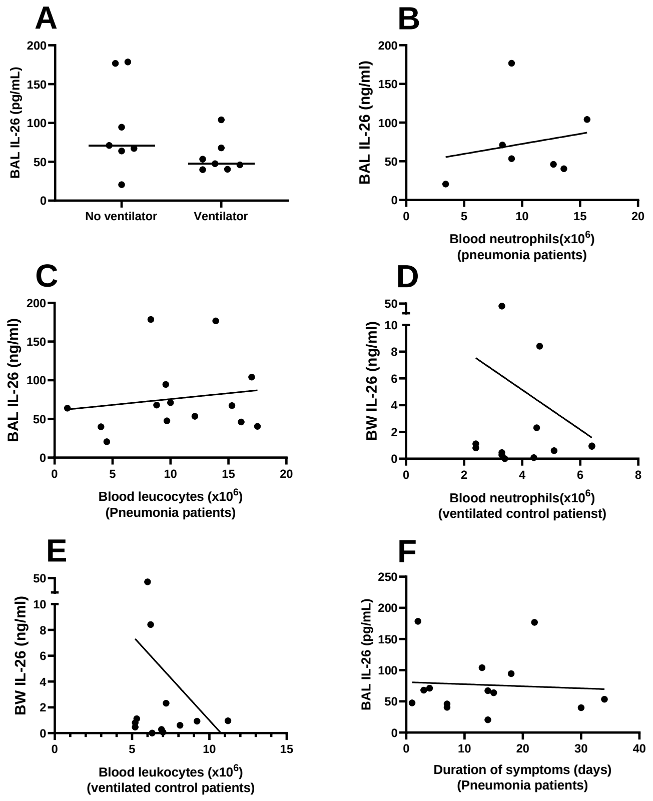

Supplementary Figure 1. IL-26 protein in the airway lumen of pneumonia patients and control subjects in relation to neutrophils, leukocyte counts and duration of symptoms. IL-26 protein concentrations were quantified in cell-free bronchoalveolar lavage (BAL) or bronchial wash (BW) samples using ELSA. **(A)** Concentrations of IL-26 in BAL of ventilated pneumonia patients (n =6), compared to unventilated patients (n =6), **(B)** concentrations of IL-26 protein in cell-free BAL fluid from pneumonia patients, in relation to blood neutrophil counts (n=7), **(C)** concentrations of IL-26 protein in cell-free BAL fluid from pneumonia patients, in relation to blood leucocyte counts (n=14), **(D)** concentrations of IL-26 protein in cell-free BW fluid from ventilated control patients, in relation to blood neutrophil counts (n=12), **(E)** concentrations of IL-26 protein in cell-free BW fluid from ventilated control

subjects, in relation to blood leucocyte counts (n=12), **(F)** concentrations of IL-26 protein in cell-free BAL fluid from pneumonia patients, in relation to duration of symptoms (n=12).

Supplemenrary Figure 2

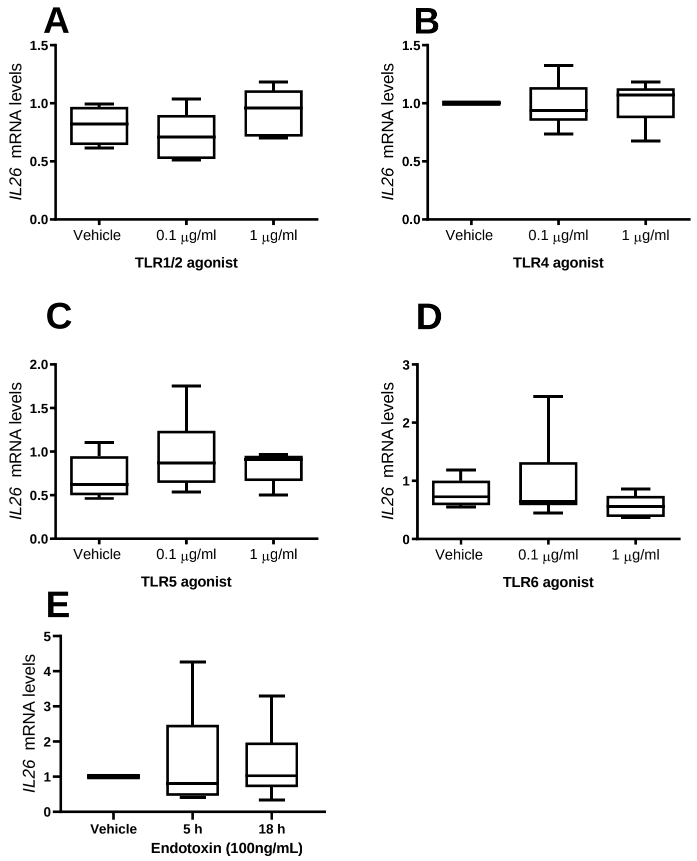

Supplementary Figure 2: IL-26 mRNA levels in type II alveolar epithelial cells exposed to toll-like receptor agonists: A human model of type II alveolar cells (A549 cells) was stimulated with toll-like receptor (TLR) agonists. Neutrophils were also stimulated with endotoxin. The cellular mRNA for IL-26 was then measured using reverse quantitative transcriptase polymerase chain reaction (qRT-PCR), (**A)** IL-26 mRNA in A549 cells, in response to the TLR1/2 agonist (Pam3CSK4) (n=6), (**B)** IL-26 mRNA in A549 cells, in response to the TLR4 agonist (endotoxin/LPS) (n=6), **(C)** IL-26 mRNA in A549 cells, in response to the TLR5 agonist (flagellin) (n=6), **(D)** IL-26 mRNA in A549 cells, in response to

the TLR6 agonist (Pam2CGDPKHPKSF) (n=6). **(E)** IL-26 mRNA in neutrophils, in response to endotoxin. Data is presented as median with range.

Supplementary Figure 3

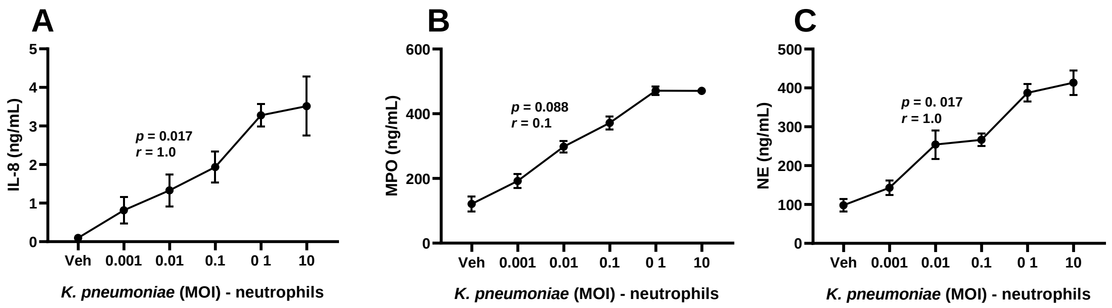

Supplementary Figure 3. Extracellular IL-26 protein concentrations in neutrophils with and without exposure to Klebsiella pneumoniae. Neutrophils were stimulated with *K. pneumoniae* at different multiplicity of infections (MOI). IL-26 protein concentrations were then quantified in the cell-free conditioned media using ELISA. **(A)** IL-8 protein concentration (*n*=12), **(B)** MPO protein concentration (*n*=12) and **(C)** NE protein concentration (*n*=12). The graphs are presented with standard error of the means (SEM) and computed according to the Spearman rank correlation test. *p*-values <0.05 are considered to indicate statistical significance.

Supplementary Figure 4

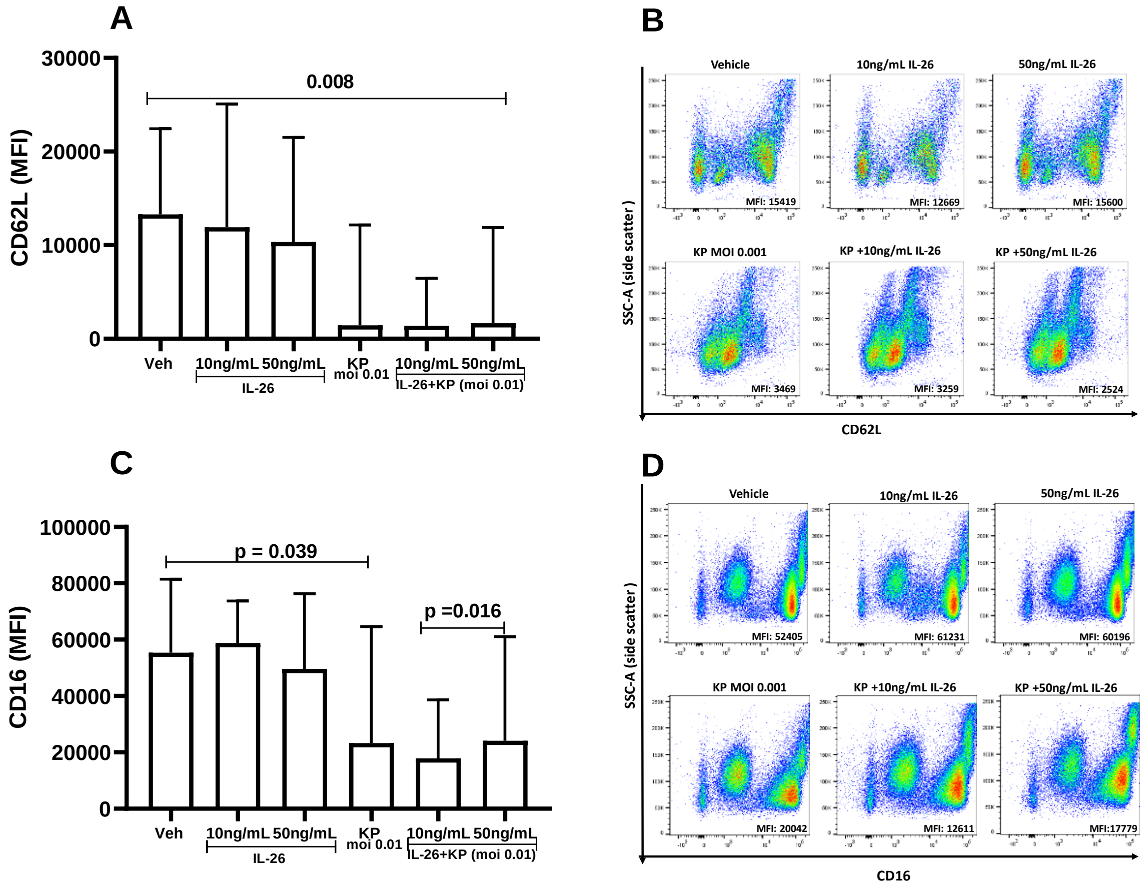

Supplementary Figure 4. Effects of exogenous IL-26 on the expression of CD62L and CD16 on neutrophils exposed to *Klebsiella Pneumoniae*. A human model of airway neutrophils (blood neutrophils) was exposed to *K. Pneumoniae* (multiplicity infection (MOI) 0.01) with and without additional stimulation by rhIL-26 (10 and 50ng/mL) for 3 hours. The cell surface markers of the state of activation were stained and measured using flow cytometry. The median florescent intensity (MFI) for each marker was then determined and plotted as graphs, **(A)** The graph shows MFI of CD62L expression for all the samples (n = 8). **(B)** Representative scatter plots for all conditions during exposure with respect to CD62L expression, **(C)** The graph shows MFI of CD16 expression for all the samples (n = 8), **(D)** Representative scatter plots for all conditions with respect to CD16 expression. The results are

presented as median with range and the *p-values* are according to the Wilcoxon Signed ranked test. Here, p-values <0.05 were considered statistically significant.

Supplementary Figure 5

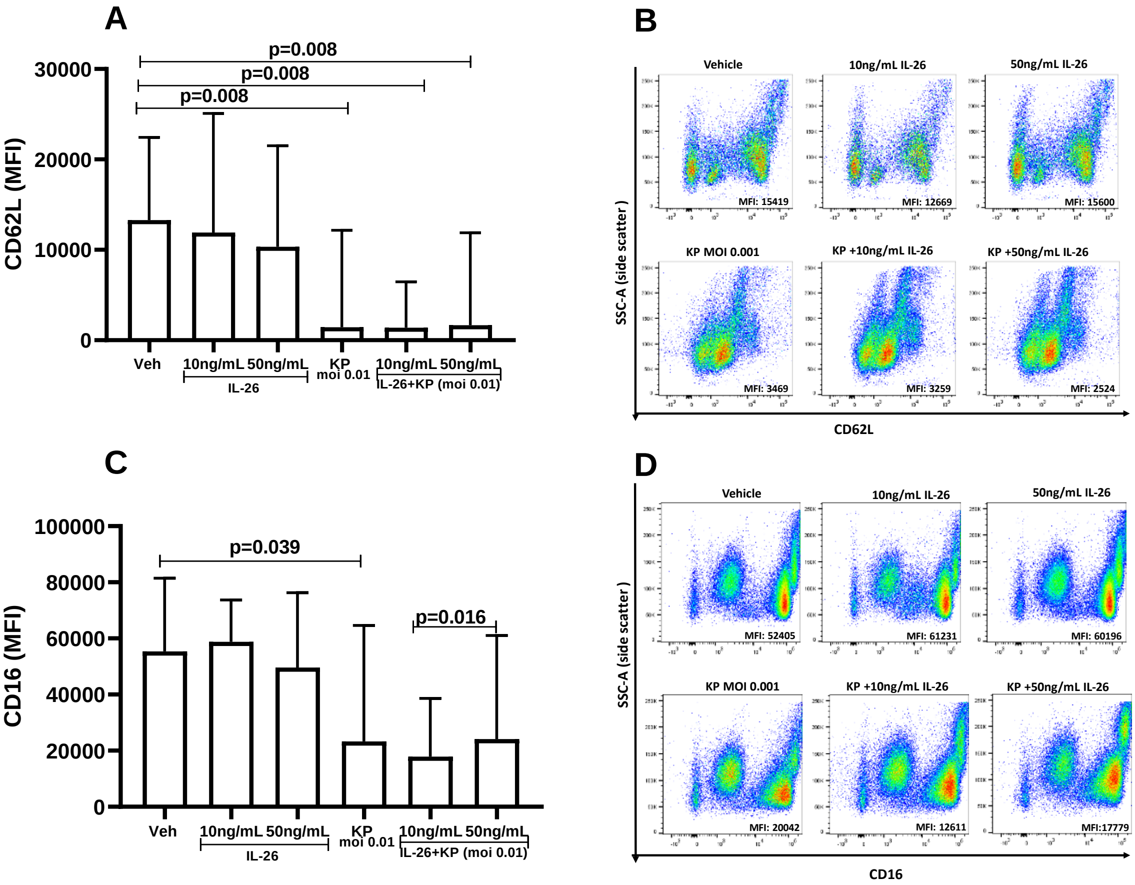

Supplementary Figure 5. Effects of exogenous IL-26 on the expression of CD49d and CD15 on neutrophils exposed to *Klebsiella Pneumoniae*. A human model of airway neutrophils (blood neutrophils) was exposed to *K. pneumoniae* (multiplicity infection (MOI) 0.01) with and without rhIL-26 (10ng/mL and 50ng/mL) for 3 hours. The cell surface markers were stained and measured using flow cytometry. The median florescent intensity (MFI) for each marker was then determined and plotted as graphs, **(A)** The graph shows MFI of CD49d expression for all the samples (n = 8). **(B)** Representative scatter plots for all conditions during exposure with respect to CD49d expression, **(C)** The graph shows MFI of CD15 for all the

samples (n = 8), (**D)** Representative scatter plots for all conditions with respect to CD15 expression. The results are presented as median with range and the *p-values* are according to the Wilcoxon Signed ranked test. The *p-values* <0.05 were considered statistically significant.

Supplementary Table 1.

**Bacterial species**	BAL	BW
Hemophilus influenzae	-	1
Pseudomonas aeruginosa	3	-
Klebsiella pneumoniae	2	-
Streptococcus pneumoniae	-	3
Mycoplasma pneumoniae	-	1
Stenotrophomonas maltophilia,	1	1
Escherichia coli,	-	1
Moraxella catarrhalis	-	1
Proteus vulgaris,	-	1
Enterococcus faecium,	2	-
Serratia marcescens,	1	-
Staphylococcus aureus	2	-
Legionella pneumophila	1	-
Nocardia farcinica,	1	-
Enterobacter cloacae	1	-
Mycobacterium avium	1	-

Supplementary Table 1. Bacteria species and their frequency of distribution. BAL: Bronchoalveolar lavage, BW: Bronchial wash sample
